# An Uncommon Cause of Dyspnea: Parsonage-Turner Syndrome With Bilateral Phrenic Nerve Involvement

**DOI:** 10.7759/cureus.115594

**Published:** 2026-09-01

**Authors:** Rajae El Kilali, Loudiyi Sara, Karima Marc

**Affiliations:** 1 Pulmonology Department, Moulay Youssef Hospital, Ibn Sina University Hospital, Rabat, MAR

**Keywords:** bilateral diaphragmatic paralysis, neuralgic amyotrophy, orthopnea, parsonage-turner syndrome, phrenic nerve palsy

## Abstract

Parsonage-Turner syndrome, also called neuralgic amyotrophy, typically affects the brachial plexus, while involvement of the phrenic nerve is far less commonly documented. When present, it can compromise diaphragmatic function, producing breathlessness with activity, orthopnea, or, in more advanced presentations, respiratory failure. These features are easily mistaken for other cardiac or pulmonary conditions, which often delays correct diagnosis.

A 51-year-old woman presented with a five-month course of worsening breathlessness on exertion and inability to breathe comfortably while supine. Cardiac work-up did not identify an underlying cause. Radiological assessment of the chest showed abnormal elevation of the right side of the diaphragm, with no associated pleural or lung tissue findings. Her history, obtained retrospectively, included an earlier episode of severe pain affecting the neck and left shoulder region, linked temporally to a stressful period. Ultrasound examination showed reduced diaphragmatic movement on both sides. Lung function testing showed a restrictive defect, and vital capacity fell markedly upon lying down compared with sitting. Electrophysiological testing identified a bilateral brachial plexopathy pattern, with phrenic nerve dysfunction predominating on the left side and additional denervation changes affecting other nerve territories, in keeping with neuralgic amyotrophy. She responded well to a combined respiratory rehabilitation approach incorporating ventilatory assistance, with subsequent improvement in her symptoms and lung volumes.

While diaphragmatic involvement occurs infrequently in neuralgic amyotrophy, it carries meaningful clinical implications and merits consideration whenever dyspnea or orthopnea cannot be explained, especially after cardiac disease is excluded and imaging identifies a raised hemidiaphragm on either or both sides. The combination of a significant postural fall in vital capacity, suggestive diaphragmatic ultrasound findings, and electrophysiological data (nerve conduction studies/electromyography) supports the diagnosis. Prompt clinical suspicion of this etiology, when relevant, allows for faster initiation of respiratory rehabilitation and ventilatory support, if required.

## Introduction

Dyspnea is one of the most common symptoms encountered in pulmonary practice, and its etiologies encompass a broad spectrum, ranging from cardiopulmonary diseases to neuromuscular disorders. Among the latter, diaphragmatic dysfunction remains an underrecognized and frequently overlooked cause, often overshadowed by more common respiratory conditions, which may lead to diagnostic delay and inappropriate management.

Parsonage-Turner syndrome, or neuralgic amyotrophy, is a rare clinical entity classically characterized by acute shoulder or upper limb pain followed by muscle weakness [1,2]. Its pathophysiology remains incompletely understood, but immunological, genetic, and mechanical mechanisms are likely involved [2].

Although the brachial plexus is predominantly affected, neuralgic amyotrophy can involve numerous peripheral nerves, including the suprascapular nerve, the long thoracic nerve, and, more rarely, the phrenic nerve [3,4].

Phrenic nerve involvement can result in diaphragmatic paresis or paralysis, most often unilateral but occasionally bilateral [3]. Its clinical presentation is highly variable, ranging from an incidental radiographic finding to significant exertional dyspnea, orthopnea, or respiratory failure.

We report a case of bilateral diaphragmatic dysfunction secondary to Parsonage-Turner syndrome, confirmed by electromyography (EMG). This case underscores the importance of considering a neuromuscular etiology, particularly diaphragmatic dysfunction, in the setting of unexplained dyspnea or orthopnea, especially when associated neurological signs are present.

## Case presentation

A 51-year-old woman initially presented to cardiology with a five-month history of progressive chronic dyspnea and orthopnea. Transthoracic echocardiography was unremarkable, and B-type natriuretic peptide levels were within normal limits.

Due to the persistence of orthopnea after a cardiac origin had been excluded and elevation of the right hemidiaphragm on chest radiography (Figure 1), the patient was referred to the pulmonology department for further management. Chest CT confirmed elevation of the right hemidiaphragm, with no pleuroparenchymal lesion (Figure 2).

**Figure 1:**
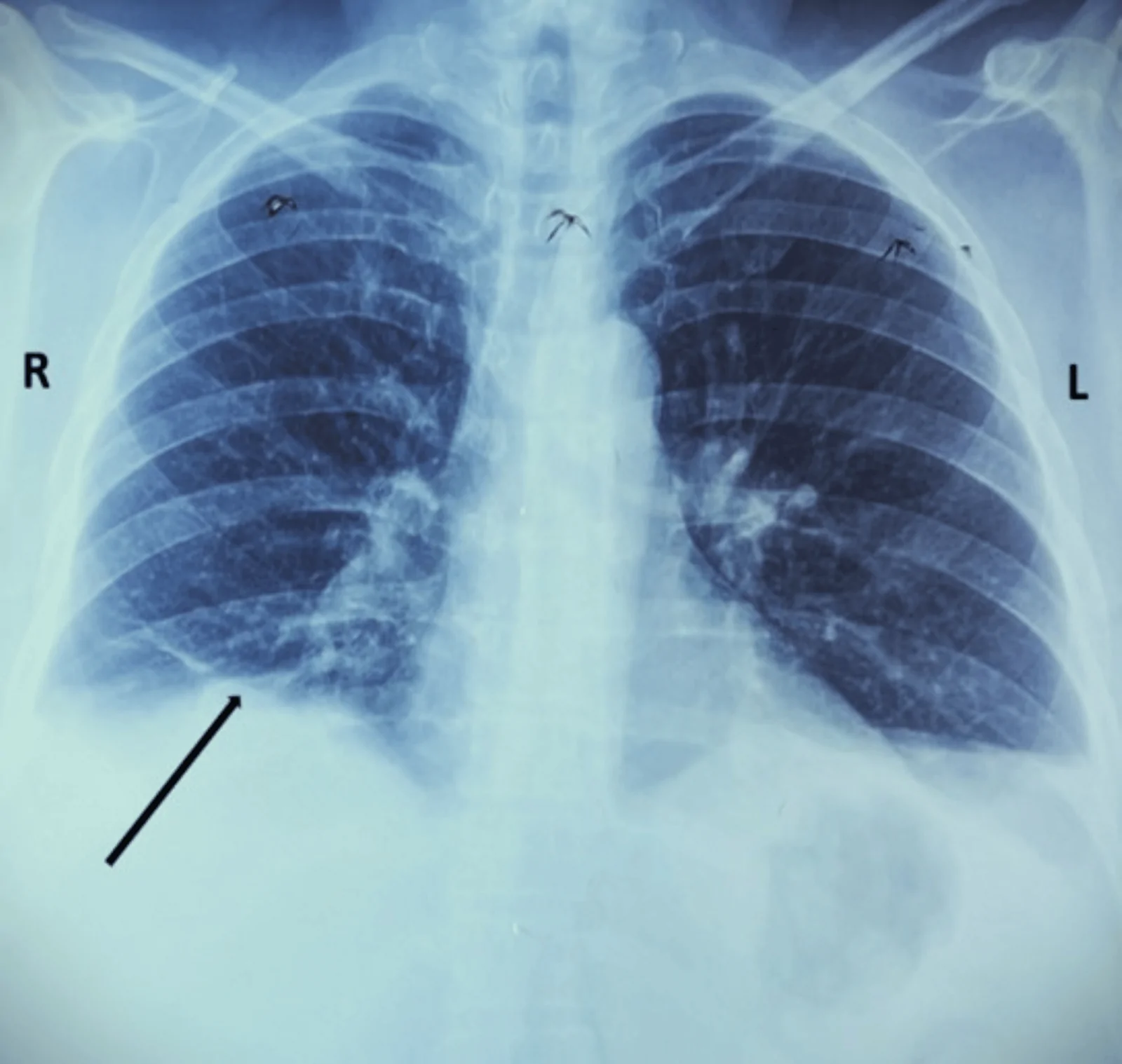
Chest radiography demonstrating elevation of the right hemidiaphragm

**Figure 2:**
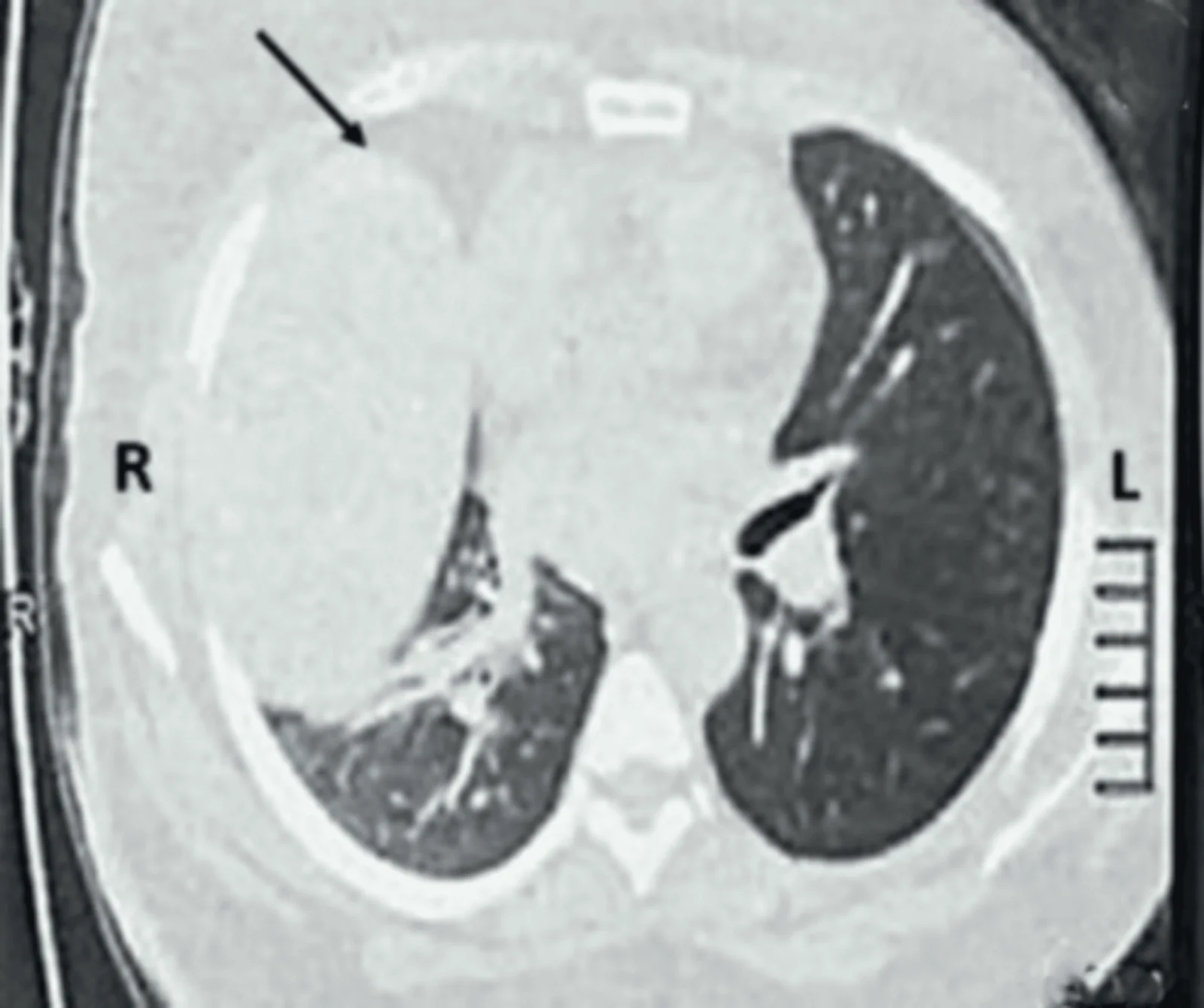
Axial CT thorax image demonstrating elevation of the right hemidiaphragm

On admission to the pulmonology department, a thorough history was taken. The patient reported that, prior to the onset of exertional dyspnea and orthopnea, she had experienced, in the context of psychological stress, the sudden onset of left-sided C4-C5 cervical neuralgia associated with severe left shoulder pain, which spontaneously resolved after two weeks, with no reported limb weakness. History-taking ruled out an iatrogenic cause, including surgery or trauma, as well as any infectious symptomatology.

Clinical examination revealed a patient with an elevated body mass index, afebrile, eupneic, with adequate oxygen saturation (SpO_2_ = 95%). Inspection revealed thoracoabdominal paradoxical movement in the supine position, without signs of respiratory distress, and auscultation revealed no crackles. The remainder of the clinical examination, including the neurological examination, was unremarkable. Diaphragmatic ultrasound showed diaphragmatic hypotonia, with a demonstrated reduction in the amplitude of its movements.

Body plethysmography identified a restrictive ventilatory pattern, with a reduced total lung capacity of 2.84 L (74% predicted), without obstructive impairment. Positional spirometry was performed to assess forced vital capacity (FVC) variability between the seated and supine positions. On assuming the supine position, FVC decreased by approximately 34% (seated FVC = 1.22 L, 52% predicted; supine FVC = 0.81 L, 35% predicted). The results of the patient's positional spirometry are summarized in Table 1.

**Table 1:** Seated and supine spirometry finding Positional spirometry demonstrated a marked reduction in FVC of approximately 34% when transitioning from the seated to the supine position, consistent with significant diaphragmatic dysfunction FEV1: forced expiratory volume in one second; FVC: forced vital capacity

Spirometry parameter	Seated position	Supine position
FEV₁, L (% predicted)	1.06 (54%)	0.68 (35%)
FVC, L (% predicted)	1.22 (52%)	0.81 (35%)
FEV₁/FVC	87%	84%

Taken together, these findings were highly suggestive of diaphragmatic dysfunction. EMG, performed to complete the work-up, revealed bilateral phrenic nerve involvement, more pronounced on the left, together with involvement of the long thoracic nerves and active denervation signs in the right deltoid muscle. These electrophysiological findings were consistent with a bilateral multitruncal brachial plexopathy, supporting the diagnosis of Parsonage-Turner syndrome (Figures 3, 4). Quantitative phrenic nerve conduction findings are summarized in Table 2.

**Figure 3:**
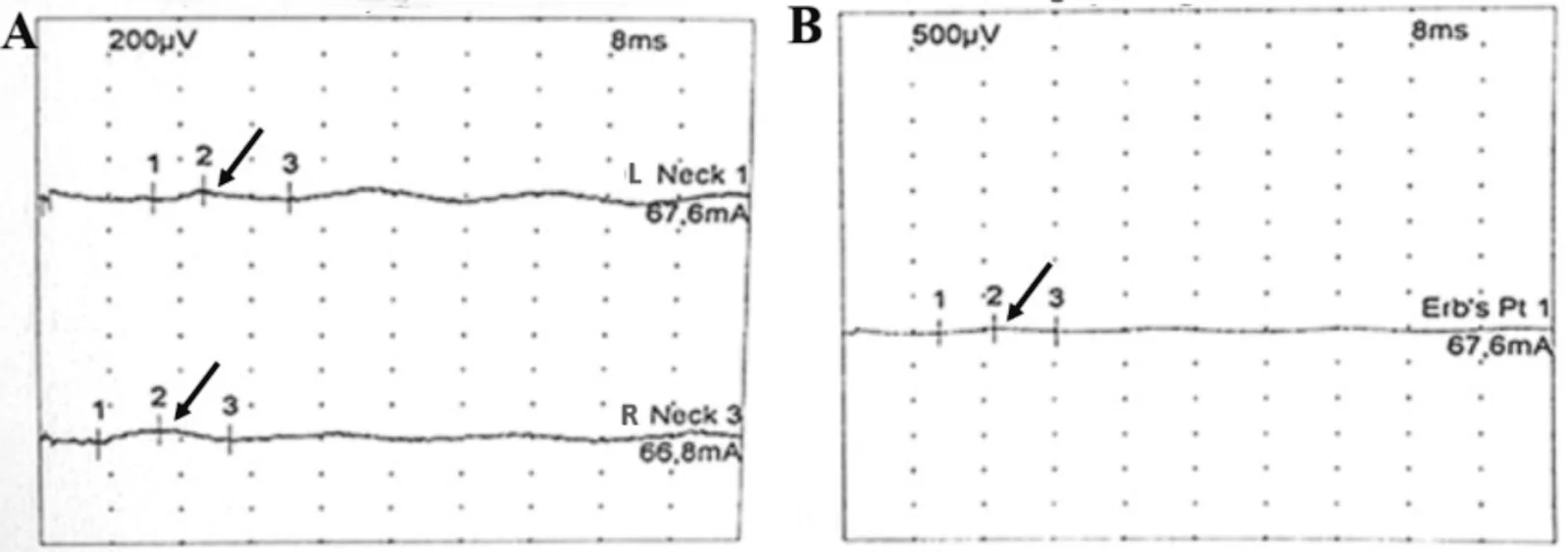
Nerve conduction studies of the phrenic and long thoracic nerves (A) Bilateral phrenic (diaphragm) nerve stimulation showing reduced motor response amplitudes, with more pronounced involvement on the left. (B) Left long thoracic (serratus anterior) nerve stimulation showing a reduced motor response amplitude. Arrows indicate the corresponding motor responses

**Figure 4:**
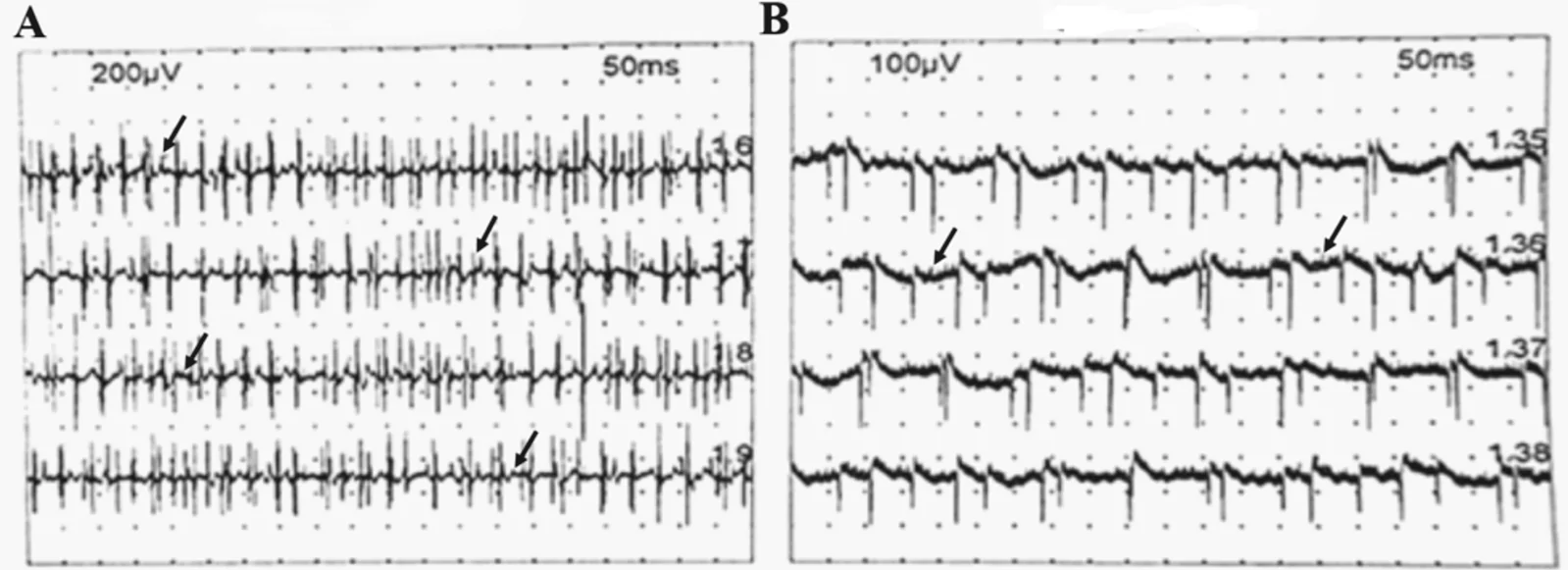
Needle electromyography of the right deltoid muscle demonstrating active denervation, with fibrillation potentials and positive sharp waves (arrows), recorded at a separate site during the same examination Recording at a sensitivity of (A) 200 µV/division and (B) 100 µV/division

**Table 2:** Quantitative findings of bilateral phrenic nerve conduction studies CMAP: compound muscle action potential

Parameter	Right phrenic nerve	Left phrenic nerve
Latency (ms)	6.72	12.97
CMAP amplitude (mV)	0.1	0.0
CMAP duration (ms)	14.84	15.31

Therapeutic management combined noninvasive ventilation (NIV) with respiratory physiotherapy, including diaphragmatic strengthening exercises. The patient reported clinical improvement, with a functional respiratory gain reflected in an increase in supine FVC to 1.02 L (44%) from an initial value of 0.81 L (35%).

## Discussion

Neuralgic amyotrophy, or Parsonage-Turner syndrome, named after the two physicians credited with its first description in a 1948 article published in The Lancet, although the condition had been described on several occasions before then [2], is an idiopathic neuropathy frequently affecting the brachial plexus. Its incidence was previously estimated at one to six cases per 100,000 person-years, with a male predominance [1]. However, a recent prospective study reported a considerably higher incidence of one case per 1,000 person-years [5]. It can occur at any age, with a median age of onset of approximately 40 years in the idiopathic form and approximately 25 years in the hereditary form [1].

The exact pathophysiological mechanism underlying this condition remains unknown but is considered complex and multifactorial. Several authors describe an interaction between environmental factors, including infections or other immune stimuli; mechanical factors, such as repetitive movements or strenuous physical exertion; and individual susceptibility, particularly genetic predisposition. Many patients report a preceding triggering event, most commonly an infection, surgery, childbirth, or physical or psychological stress [2]. In the study by Tsairis et al. [6], which included 99 cases of Parsonage-Turner syndrome, 25 patients reported a preceding flu-like illness. Our patient, in contrast, reported psychological stress preceding the onset of her acute symptoms.

Clinically, the condition classically begins acutely, with sudden and intense scapular pain, often occurring at night, typically lasting two to three weeks and responding poorly to usual analgesics. This condition is often initially mistaken for glenohumeral bursitis or a simple muscle strain, which frequently accounts for a diagnostic delay that can extend to several months [2].

Although recognized, phrenic nerve involvement remains a rare manifestation of neuralgic amyotrophy, reported in approximately 7.6% of patients in one published cohort, and may result in unilateral or bilateral diaphragmatic paralysis [3]. Previous observations have also shown that this involvement may present primarily as dyspnea or orthopnea, leading to diagnostic delay when more common cardiopulmonary causes are investigated first [4,7].

Diaphragmatic ultrasound also constitutes a useful noninvasive examination for assessing diaphragmatic movement. Chest radiography and CT most often reveal elevation of one or both hemidiaphragms and help rule out a mediastinal lesion compressing the phrenic nerve, while MRI, not routinely performed, allows for the exclusion of an associated cervical radiculopathy.

Pulmonary function tests typically show a restrictive pattern. Diaphragmatic dysfunction is suggested when the fall in FVC between the upright and supine positions exceeds 20%-30%. This feature was observed in our patient, with a 34% decrease in FVC [8].

The indication for initiating NIV in patients with neuromuscular disease is based on a daytime partial pressure of carbon dioxide greater than 45 mmHg, oxygen saturation below 88% for more than five minutes during the night, or an FVC below 50% predicted. These indications can be extended to patients with diaphragmatic dysfunction, in whom NIV should also be offered. This was the case in our patient.

EMG cannot rule out a diagnosis of neuralgic amyotrophy: nerve conduction studies are often normal, and needle EMG detects abnormalities only when the muscles that are actually affected are tested, which is rarely the case in practice. The diagnosis therefore remains largely clinical [2]. However, in our patient, EMG confirmed the clinical diagnosis by demonstrating bilateral multitruncal involvement of the brachial plexus.

An apparent discrepancy was observed between the imaging and electrophysiological findings. Chest radiography and CT showed right-sided hemidiaphragmatic elevation, whereas electrophysiological studies demonstrated bilateral phrenic nerve involvement, more pronounced on the left. This apparent discrepancy may be related to the multifocal and potentially asymmetric nature of neuralgic amyotrophy, in which phrenic nerve involvement can be unilateral or bilateral [3]. Moreover, imaging and electrophysiological studies provide complementary information: static imaging primarily assesses diaphragmatic position and morphology, whereas electrophysiological testing evaluates phrenic nerve function. In our patient, the right-sided radiological abnormality contrasted with the greater left-sided electrophysiological impairment, highlighting the value of combining imaging, diaphragmatic ultrasound, and electrophysiological assessment when evaluating suspected phrenic neuropathy.

Early-stage treatment generally relies on analgesia and, in some cases, corticosteroid therapy, although published data have not shown a clear benefit of corticosteroids on the recovery of respiratory function [6]. Respiratory physiotherapy, including diaphragmatic strengthening exercises, optimization of any underlying respiratory disease, and functional rehabilitation, remains the mainstay of management.

Recovery is usually spontaneously favorable, with two-thirds to three-quarters of patients recovering within two years. In contrast, the prognosis of diaphragmatic involvement appears less favorable. In a study by Hughes et al. [9] involving 14 patients with bilateral diaphragmatic paralysis, the mean recovery time was 68 months. Complete recovery, with normalization of inspiratory pressures, was observed in only two cases. Recovery was partial in eight patients and absent in the remaining four.

In a more recent case series of four patients with unilateral diaphragmatic paralysis secondary to Parsonage-Turner syndrome with phrenic nerve involvement, complete recovery at 18 months was observed in one patient, while two patients remained symptomatic at six months. The fourth patient reported partial clinical improvement three years after diagnosis, without complete recovery of his previous functional status. All patients remained under follow-up [10].

In our patient, clinical and functional improvement was observed after one month, and she is being followed regularly at our institution.

## Conclusions

Parsonage-Turner syndrome is a rare and multifactorial neurological disorder involving genetic, biomechanical, and immunological factors that remains frequently underrecognized in clinical practice. This diagnosis should be considered in any patient with persistent orthopnea that remains unexplained after a noncontributory cardiac work-up, particularly when preceded by a viral infection, periscapular or upper limb pain, or a neurologically-mediated motor deficit.

The combination of this clinical picture with unilateral or bilateral hemidiaphragm elevation on chest radiography, a marked decline in pulmonary volumes in the supine position on spirometry, and electromyographic evidence of neurological involvement provides a decisive set of findings to confirm the diagnosis. Early recognition of this syndrome may help reduce diagnostic delay and guide appropriate management, based primarily on rehabilitation, diaphragmatic strengthening, and, in cases of nocturnal alveolar hypoventilation, NIV, with regular follow-up given the risk of recurrence.
